# Neurotropic alphaviruses can propagate without capsid

**DOI:** 10.18632/oncotarget.13993

**Published:** 2016-12-16

**Authors:** Marta Ruiz-Guillen, Nicola G.A. Abrescia, Cristian Smerdou

**Affiliations:** Division of Gene Therapy, CIMA, University of Navarra, Pamplona, Spain; and IdiSNA, Navarra Institute for Health Research, Pamplona, Spain; Structural Biology Unit, CIC bioGUNE, CIBERehd, Bizkaia Technology Park, Derio, Spain; IKERBASQUE, Basque Foundation for Science, Bilbao, Spain

**Keywords:** alphavirus, semliki forest virus, sindbis virus, capsid-deficiency, infectious microvesicles, Neuroscience

Alphaviruses are a group of enveloped viruses transmitted by mosquitoes. Many alphaviruses are neurotropic, being able to produce encephalitis in humans and animals. Among alphaviruses, both Semliki Forest (SFV) and Sindbis virus (SIN) have been extensively studied as models of viral pathogenicity. In mice, SFV and SIN can infect neurons in the central nervous system and virulent strains induce lethal encephalitis, while avirulent SFV strains induce demyelination [1]. These viruses contain an icosahedral nucleocapsid formed by 240 capsid monomers packaging a positive-strand RNA genome of around 12 kb. The nucleocapsid is surrounded by a lipid envelope coated by viral envelope proteins E1 and E2, or spikes, required for infection. SFV has been used as a model enveloped virus to study different steps in viral infection, including entry, endosomal release, and budding. In fact, SFV was used by Ari Helenius (ETH Zurich, Switzerland) to describe the mechanism by which most enveloped viruses are released from endosomes in infected cells [2]. This process, essential for many human pathogenic viruses like hepatitis C virus (genus Hepacivirus) or Ebola virus (genus Filovirus), is mediated by conformational changes in viral envelope proteins induced by acidic endosomal pH, leading to fusion of viral and endosome membranes, and resulting in cytosolic release of the viral genome [3]. As described for other positive-strand RNA viruses, alphavirus genomes replicate in association with intracellular membranes, leading to very high expression of capsid and spike proteins. Viral budding requires the interaction of pre-assembled nucleocapsids with cytoplasmic domains of viral spike proteins [4]. Although this last process is considered essential in the virus life cycle, we have recently demonstrated that alphavirus budding and propagation is also possible in the absence of capsid protein [5]. This phenomenon, observed for both SFV and SIN, takes place because cells in which capsid-less alphavirus genomes are replicating generate, at the plasma membrane, microvesicles (iMVs) coated by spike proteins capable to uptake viral genomic RNAs, rendering them infectious (Figure 1, left). Interestingly, iMVs use the endocytic pathway described by A. Helenius for wild type (wt) SFV to infect cells (Figure 1, right). A detailed characterization of iMVs showed that they are pleomorphic, possess a larger size than wtSFV, are able to package small amounts of other viral and cellular RNAs, and are not derived from exosomes or viral replication complexes [5]. A similar type of iMVs had been previously described by the group of John K. Rose (Yale University, CT) when they used a SFV vector devoid of all viral structural genes to express vesicular stomatitis virus G protein [6]. This chimeric vector was also able to propagate in cell culture and has been successfully used in several vaccination studies.

**Figure 1:**
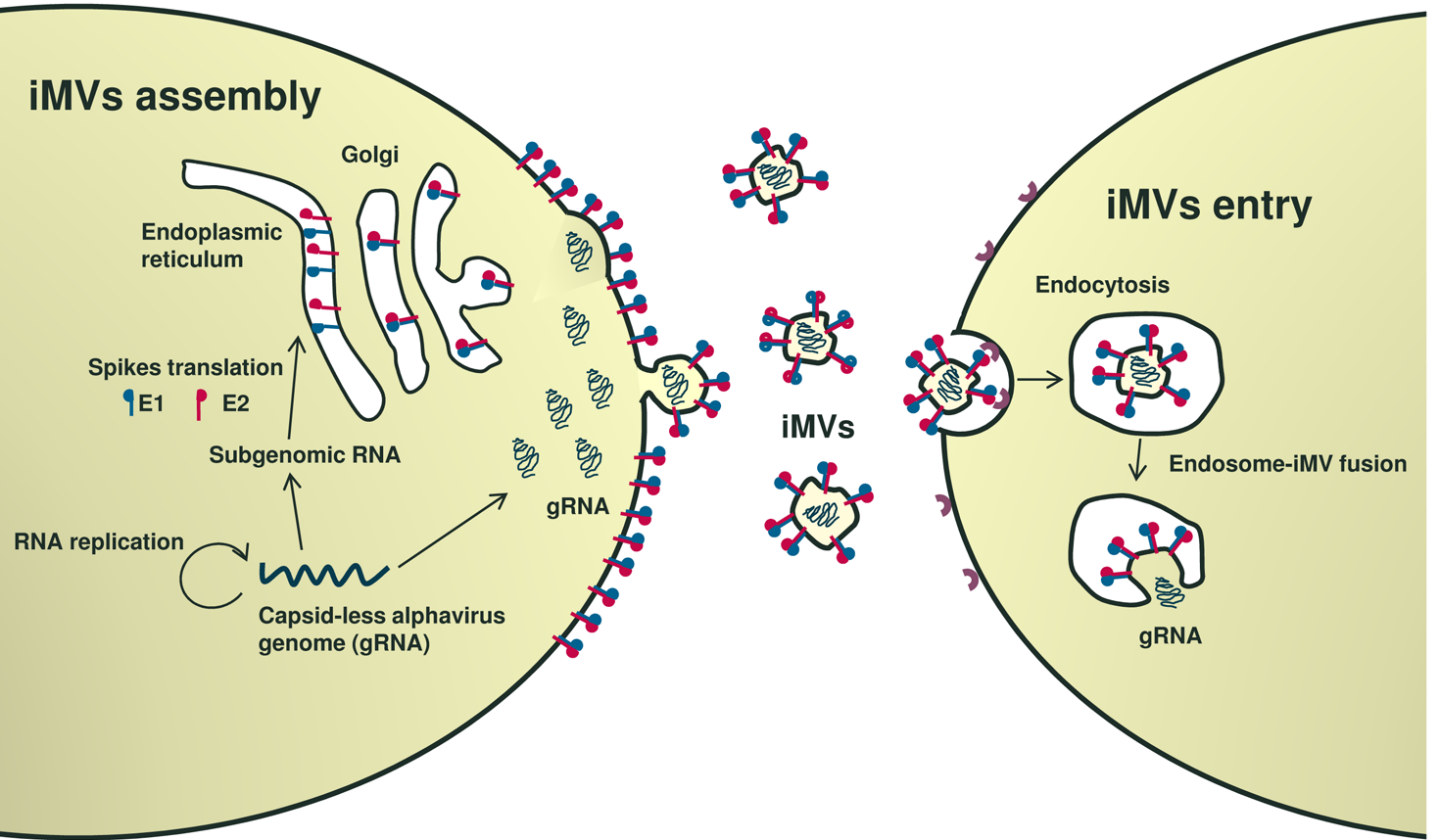
Propagation of capsid-less alphavirus

Given the neurotropic nature of wtSFV, we investigated whether SFV iMVs could also infect the brain and induce disease in mice. Interestingly, iMVs were able to infect some organs, such as lungs and heart, when administered systemically. However, they did not reach the brain and did not induce any observable pathology, in contrast to wtSFV, which was detected in brain and killed 60% of mice a few days after infection [5].

These results suggested that iMVs could be potentially used for *in vivo* treatments that include vaccination or gene therapy. In this regard it will be interesting to test whether highly pathogenic alphaviruses, like Venezuelan equine encephalitis virus or Chikungunya virus, can also generate this kind of iMVs. If this is the case, iMVs derived from these viruses may potentially serve as “attenuated” vaccines for human use. In contrast to vaccines based on empty virus-like particles, iMVs, by replicating and expressing viral spike proteins *in vivo*, could in principle potentiate antiviral immune responses.

Moreover, we have shown that it is possible to insert foreign genes into the SFV genome devoid of capsid, leading to spreading of transgene expression in cell culture through the generation of “recombinant” iMVs. However, one potential drawback for the use of iMVs is that they are generated at very low titers, approximately 10^4^-fold lower than those obtained for wtSFV. This issue can be partially overcome through “packaging” the capsid-less SFV genome into conventional viral particles by providing the capsid gene in *trans* [5]. Viral particles obtained in this way behave like wtSFV when they infect cells, but then are able to propagate by producing iMVs, since the capsid gene is no longer present. We have tested this approach to generate a propagative vector expressing interleukin-12 (IL-12), a cytokine with strong antitumor properties [7]. When used at a low multiplicity of infection, this vector was able to propagate, leading to higher IL-12 expression levels than a conventional non-propagative SFV vector *in vitro*. Notably, this propagative vector induced potent antitumor responses in a mouse model of colorectal cancer (unpublished data), suggesting that it could have potential use for tumor gene therapy.

Recently, the group of Ari Hinkkanen (University of Eastern Finland, Kuopio, Finland) has also revealed that a virulent strain of wtSFV (SFV4) can function as an efficient oncolytic agent against orthotopic gliomas in mice [8]. In this case, and in order to reduce virus toxicity, the SFV4 genome was modified by including microRNA target sequences that limit replication in healthy neurons. Whether the iMVs described by us, also derived from SFV4 but showing no toxicity in mice, are able to mediate similar oncolytic effects by direct administration into brain tumor models remains a hypothesis to be tested.

In summary, while infectious iMVs challenge our view on the assembly of alphavirus they open a new scenario of potential applications in cancer biology, vaccinology and neuroscience.

## References

[1] Atkins GJ (1999). J Gen Virol.

[2] Helenius A (1980). J Cell Biol.

[3] Harrison SC (2015).

[4] Suomalainen M (1992). J Virol.

[5] Ruiz-Guillen M (2016). Cell Mol Life Sci.

[6] Rose NF (2014). Proc Natl Acad Sci U S A.

[7] Quetglas JI (2013). J. Immunol.

[8] Martikainen M (2015). J Virol.

